# Exploring the Associations between Chronotype, Night Shift Work Schedule, Quality of Work Life, and Sleep Quality among Maternal and Child Health Nurses: A Multicentre Cross-Sectional Study

**DOI:** 10.1155/2023/1811732

**Published:** 2023-09-30

**Authors:** Jia-Ning Li, Xiao-Qian Chen, Xiu-Min Jiang, Qing-Xiang Zheng, Yu-Qing Pan, Yu Zhu, Ling Huang, Ru-Lin Liu

**Affiliations:** ^1^School of Nursing, Fujian Medical University, Fuzhou, Fujian, China; ^2^Fujian Maternity and Child Health Hospital College of Clinical Medicine for Obstetrics & Gynecology and Pediatrics, Fujian Medical University, Fuzhou, Fujian, China; ^3^Fujian Obstetrics and Gynecology Hospital, Fuzhou, Fujian, China; ^4^School of Nursing, Fujian University of Traditional Chinese Medicine, Fuzhou, Fujian, China

## Abstract

**Aim:**

To examine the state of sleep quality among maternal and child health (MCH) nurses and explore the associations between chronotype, night shift work schedule, quality of work life, and sleep quality among MCH nurses.

**Background:**

MCH nurses, who play an important role in protecting the health of women and children, often experience poor sleep quality. However, research on the sleep quality of MCH nurses has been scarce following implementation of the three-child policy in China.

**Methods:**

A multicentre cross-sectional study was conducted with 1426 MCH nurses. Data were collected using a demographic questionnaire, participants' self-reported chronotype, the Pittsburgh Sleep Quality Index, and the Work-Related Quality of Life-2 scale. A chi-squared test, independent samples *t*-test, Pearson correlation test, and binary logistic regression analysis were used to analyse the data.

**Results:**

Of the 1426 respondents, 57.9% reported poor sleep quality. Binary logistic regression analysis indicated that chronotype, including intermediate-morning, intermediate-evening, and evening (reference: morning), and quality of work life, including stress at work, control at work, and general well-being, had effects on sleep quality among MCH nurses. Older age, frequent caffeine intake, and irregular meals were also associated with poor sleep quality. However, night shift work schedule did not affect sleep quality in the adjusted model.

**Conclusions:**

Poor sleep quality was common among MCH nurses. The findings of this study also illustrate that chronotype and quality of work life are closely related to sleep quality. *Implications for Nursing Management*. Nursing managers should be aware of MCH nurses' chronotype and quality of work life and tailor interventions to address both modifiable and nonmodifiable factors associated with sleep to improve MCH nurses' sleep quality.

## 1. Background

The health of women and children is an important global concern and an indicator of people's health on a national level [1]. According to the People's Republic of China's National Health Commission, in 2022, China's maternal mortality ratio had dropped to 15.7 deaths per 100,000 people, while infant mortality had dropped to 4.9 deaths per 1000 people [2], owing to the efforts of maternal and child health (MCH) workers [3]. As the largest professional group of MCH workers, MCH nurses play a vital role in safeguarding the health of women and children. Women and children are generally considered a vulnerable population. Physically, women require specialised care because of pregnancy and the possibility of gynaecological disorders [1]. Children require extra care and attention because they are immature and less able than adults to adapt to their surroundings [4]. Psychologically, women and children are susceptible to social and family pressures and have complex psychological needs that require care and comfort [5, 6]. Therefore, MCH nurses need compassion, concentration, and expertise in their work to meet the needs of these special groups and provide the best possible nursing care. Furthermore, children are prone to emergencies at night due to their young age and frailty [7], and nighttime births are highly likely [8]; therefore, MCH nurses need to be alert and provide constant observation and care during night shifts. The nighttime environment and work intensity can negatively affect MCH nurses' sleep quality. Furthermore, China's “three-child” policy has led to increasing rates of pregnancies in women of advanced reproductive age or repeated caesarean deliveries [9], exacerbating the shortage of MCH nurses. Based on these conditions, MCH nurses in China may bear the burden of increased workloads, resulting in poor sleep quality. Therefore, nursing managers should pay close attention to this issue.

Sleep quality is a crucial health indicator, reflecting self-perceived levels of satisfaction with sleep [10]. However, people are increasingly reporting complaints of poor sleep quality and its negative impact on daytime functioning [11]. Poor sleep poses numerous risks to individual health, including cognitive impairment, chronic fatigue, metabolic syndrome, cardiovascular disease, and hormonal disease [12–14]. Poor sleep has become a widespread issue among medical staff. Over half of nurses and midwives have reported severe or very severe daytime dysfunction [15]. Moreover, research has shown that between 32.18 and 64.5% of MCH nurses experience poor sleep [16–18], which is associated with increased rates of burnout (59.71%) and health-risk stress (54.3%) [19, 20]. Furthermore, poor sleep can affect the of MCH nurses' professional performance and increase their risk of errors and accidents, which can cause patients to feel frustrated and uncomfortable [21, 22]. Higher staff turnover caused by sleep problems negatively affects the functioning of MCH hospitals, which can directly and indirectly affect the health of women and children. In addition, research shows that sleep quality is closely associated with physical, psychological, environmental, occupational, social, and behavioural factors [23, 24]. Dissonance between an individual's chronotype and shift work schedules may be the mechanism responsible for poor sleep. Quality of work life may also affect the occurrence of sleep issues because it is a multidimensional definition that reflects well-being and quality of life in the workplace. Work-related factors, such as shift work schedules, are arranged by nursing administrative staff and deserve further exploration. Previous research has also noted the impact of work schedules involving 16-hour night shifts, 12.5-hour night shifts, and three-shift rotations on sleep quality in Japan [25]. However, the prevalence of poor sleep among MCH nurses remains unclear. No extensive research has been conducted on whether chronotype, night shift work schedule, and quality of work life affect sleep quality. In response to these gaps in the literature, this study aimed to investigate the associations between chronotype, night shift work schedule, and quality of work life with sleep quality among MCH nurses.

## 2. Materials and Methods

### 2.1. Setting and Sampling

This study used a cross-sectional survey and followed the Strengthening the Reporting of Observational Studies in Epidemiology (STROBE) guidelines. The sample size was estimated using the single population proportion formula: *N* = (*Zα*/2)^2^ × *p*(1 − *p*)/*d*^2^. According to a meta-analysis, the prevalence of poor sleep among Chinese nurses is 49.9% [26]. Given this criterion (α = 0.05;  d = 0.03), 1068 participants were required. We ultimately decided on a minimum sample size of 1187 after considering a 10% nonresponse rate. The following inclusion criteria were used: MCH nurses who have (a) obtained professional qualification certificates and (b) agreed to participate in the study. The exclusion criteria were as follows: (a) retired nurses, refresher nurses, and student nurses; (b) less than 6 months of work experience; and (c) missing more than one month of work due to illness, marriage, maternity, or other personal matters. Cluster sampling was used to recruit the study participants. Based on the annual reported number of nurses in the province, four MCH hospitals were required to meet the sample size requirements. First, convenience sampling was used to select four MCH hospitals in Fujian province. Second, all MCH nurses working at those hospitals who met the inclusion and exclusion criteria were recruited for the study between December 2022 and January 2023.

### 2.2. Measures

#### 2.2.1. Demographic Questionnaire

A demographic questionnaire was designed by the researchers based on a literature review. Personal variables included age, height, weight, sex, educational level, marital status, number of children, duration of mobile phone use before bedtime, frequency of irregular meals, frequency of caffeine intake, frequency of sugar intake, and physical activity. Physical activity was assessed using the short version of the International Physical Activity Questionnaire (IPAQ-SF). The Chinese version of the IPAQ-SF has been validated among Chinese university students [27] and measures the intensity, frequency, and duration of physical activity over the last seven days. According to the guidelines for data processing and analysis of the IPAQ [28], participants' physical activity levels were categorized into three groups: low, moderate, and high.

Work-related variables included personal monthly income, department, professional rank, employment type, and night shift work schedule. Participants' night shift work schedules were classified as non-night shift, forward-rotating night shift (i.e., evening shift between 16:00 and 24:00 h and night shift between 00:00 and 08:00 h the next day), backward-rotating night shift (i.e., night shift between 00:00 and 08:00 h and evening shift between 16:00 and 24:00 h the next day), and 12-hour rotating night shift (i.e., night shift between 20:00 and 08:00 h).

#### 2.2.2. Self-Reported Chronotype

Chronotype reflects an individual's preferred time of day for an activity/rest cycle. Chronotypes were assessed using a subjective question asking participants to self-classify themselves by chronotype. Individuals were classified according to the following chronotypes: morning (“definitely morning person”), intermediate-morning (“more a morning than an evening person”), intermediate-evening (“more an evening than a morning person”), and evening (“definitely evening”). Self-reported chronotypes have been validated using self-reported sleep-wake times [29].

#### 2.2.3. The Pittsburgh Sleep Quality Index (PSQI)

The PSQI is a 19-item self-report instrument that measures sleep quality during the last month [30]. The PSQI consists of seven components: subjective sleep quality, sleep latency, sleep duration, sleep efficiency, sleep disturbance, sleep medication, and daytime dysfunction. Each component included one or more items scored on a scale ranging between 0 and 3 points, where 0 indicated “normal”, 1 indicated “mild dysfunction”, 2 indicated “moderate dysfunction”, and 3 indicated “severe dysfunction”. The scores of the seven components were summed to obtain a global score ranging between 0 and 21, with higher scores indicating poorer sleep quality. The Chinese version of the PSQI demonstrates good overall reliability (*r* = 0.82–0.83) and test-retest reliability (*r* = 0.77–0.85) [31]. In addition, a cutoff point of seven could effectively differentiate between good and poor sleep quality in the Chinese population [32]. Therefore, the poor sleep group was defined as participants with a PSQI total score >7, while the good sleep group was defined as participants with a PSQI total score ≤7. In this study, Cronbach's *α* coefficient for this scale was 0.752.

#### 2.2.4. The Work-Related Quality of Life-2 Scale (WRQOL-2)

The WRQOL-2 is a 34-item instrument used to assess quality of working life and was developed by Van Laar et al. and translated into Chinese and modified by Shao et al. [33, 34]. The WRQOL-2 measures seven dimensions: working conditions (WCS), stress at work (SAW), control at work (CAW), home-work interface (HWI), employment evaluation of nurses (EEN), general well-being (GWB), and job and career satisfaction (JCS). Respondents indicated their level of agreement for each dimension on a five-point scale. Furthermore, a reverse scoring method is employed for the SAW dimension. Higher scores signified a higher quality of nursing work life. The Chinese version has demonstrated good psychometric properties, with previous research reporting Cronbach's *α* coefficients of 0.939 for the total scale and between 0.652 and 0.859 for each dimension [33]. In this study, Cronbach's *α* coefficient was 0.960 for the total scale and ranged between 0.744 and 0.931 for each dimension.

### 2.3. Data Collection

Permission was obtained from the nursing department directors at each of the four hospitals prior to the study. The researchers conducted a uniform training session for the four investigators from the sample hospitals and presented the study protocol. Data were collected via WenJuanXing (a professional questionnaire survey platform). After obtaining informed consent from the participants, the investigators explained the method for completing the questionnaires to the participants using questionnaire guidance language. The participants were guaranteed anonymity and confidentiality and could withdraw freely from the study at any time and for any reason.

### 2.4. Statistical Analyses

IBM SPSS version 27.0 was used for all data analyses, and GraphPad Prism version 9.0 was used to prepare the graphs. All the data were found to be approximately normally distributed. Continuous variables were presented as mean ± standard deviation (M±SD), and categorical variables were presented as frequencies and percentages. A chi-square test and independent samples *t*-test were used to identify statistically significant differences in demographic characteristics, night shift work schedule, chronotype, and quality of work life scores between the good sleep and poor sleep groups. Pearson's correlation test was used to examine the correlation between quality of work life and sleep quality. A binary logistic regression analysis model was used to calculate odds ratios (ORs) and 95% confidence intervals (95% CIs) for poor sleep quality (PSQI > 7), which was adjusted for potential confounding variables (univariate correlates: age, educational level, duration of mobile phone use before bedtime, frequency of irregular meals, frequency of caffeine intake, personal monthly income, department, professional rank, and employment type). The model was verified using the Hosmer–Lemeshow goodness-of-fit test. All tests were two-sided, and a *P* value <0.05 was considered statistically significant.

### 2.5. Ethical Considerations

This study was approved by the Ethics Committee of the main researcher's hospital (No. 2022YJ071). All participants took part in the study voluntarily and could withdraw at any time. All methods in this study were performed according to the relevant guidelines and regulations.

## 3. Results

### 3.1. Participant Characteristics

Among the 1510 questionnaires, 1426 valid questionnaires were obtained after removing those with apparent logical errors, representing an effective response rate of 94.4%. The mean participant age was 30.20 (SD = 7.60), ranging between 20 and 60 years. Participants' average BMI was 20.95 (SD = 2.70) kg/m^2^. Most participants were female (*n* = 1401, 98.2%), married (*n* = 766, 53.7%), graduated from junior college (*n* = 754, 52.9%), had no children (*n* = 725, 50.8%), used their mobile phones for 1–3 hours before bedtime (*n* = 753, 52.8%), had irregular meals 1-2 times/week (*n* = 821, 57.6%), had a caffeine intake frequency of 3–5 times/week (*n* = 787, 55.2%), had a sugar intake frequency of 3–5 times/week (*n* = 972, 68.2%), engaged in a moderate level of physical activity (*n* = 634, 44.5%), were senior nurses (*n* = 604, 42.4%), and worked as contract employees (*n* = 952, 66.8%; Table 1).

### 3.2. Sleep Quality among MCH Nurses

In the current study, good and poor sleep quality were noted in 600 (42.1%) and 826 (57.9%) MCH nurses, respectively. PSQI global scores ranged from 0 to 21, with a mean of 7.90 (SD = 3.67). Regarding the components of PSQI, 710 (49.8%) of the respondents reported that their subjective experience of sleep quality was fairly good. Regarding sleep latency, 487 (34.2%) participants had mild difficulties falling asleep. In terms of sleep duration, 604 (42.4%) of the respondents reported sleeping for 6–7 hours per night. For sleep efficiency, 733 (51.4%) participants scored higher than 85%. There were 860 (60.3%) respondents with mild sleep disturbance, and 1222 (85.7%) had not used sleep medication in the past month. Regarding daytime dysfunction, 475 (33.3%) of the respondents had mild dysfunction (Table 2). Across all seven components, the highest scores were observed for sleep latency, followed by subjective sleep quality and daytime dysfunction. Sleep medication had the lowest scores (Table 1).

### 3.3. Personal and Work-Related Factors Associated with Sleep Quality

Regarding personal factors, sleep quality was significantly related to age, educational level, duration of mobile phone use before bedtime, frequency of irregular meals, and frequency of caffeine intake (*P*  <  0.05). For work-related factors, sleep quality was significantly correlated with personal monthly income, department, professional rank, and employment type (*P*  <  0.05; Table 1).

### 3.4. Chronotype and Night Shift Work Schedule Associated with Sleep Quality

**Figure 1:**
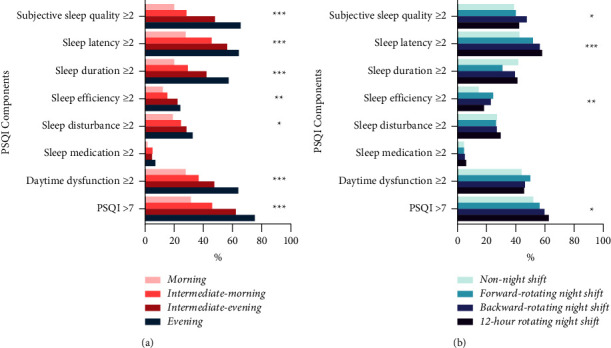
Rates of poor sleep components on each group classified by chronotype and night shift work schedule (*n* = 1426). ^*∗*^*P* < 0.05. ^*∗∗*^*P* < 0.01. ^*∗∗∗*^*P* < 0.001. (a) Rates of poor sleep components on each chronotype. (b) Rates of poor sleep components on each night shift work schedule.

Anintermediate-evening chronotype (*n* = 743, 52.1%) was the most prevalent among participants, followed by an intermediate-morning chronotype (*n* = 341, 23.9%), evening chronotype (*n* = 227, 15.9%), and morning chronotype (*n* = 115, 8.1%). Participants with an evening chronotype (*n* = 171, 75.33%) were the most likely to experience poor sleep problems, followed by those with an intermediate-evening chronotype (*n* = 462, 62.2%), intermediate-morning chronotype (*n* = 157, 46.0%), and morning chronotype (*n* = 36, 31.3%). Significant differences were observed between poor sleep and chronotype (*P*  <  0.001). When each component of sleep quality was divided into two categories, a chi-square test suggested that chronotype was associated with subjective sleep quality, sleep latency, sleep duration, sleep efficiency, and daytime dysfunction (*P*  <  0.05; Figure 1(a)).

Among the participants, 376 (26.4%) worked non-night shifts, 110 (7.7%) worked forward-rotating night shifts, 704 (49.4%) worked backward-rotating night shifts (the largest proportion of MCH nurses), and 236 (16.5%) worked 12-hour rotating night shifts. Over 50% of participants reported poor sleep for each of the night shift work schedules. MCH nurses working 12-hour rotating night shifts had the highest rate of poor sleep (*n* = 148, 62.7%), followed by those working backward-rotating night shifts (*n* = 420, 59.7%), forward-rotating night shifts (*n* = 62, 56.4%), and non-night shifts (*n* = 196, 52.1%). Significant differences in poor sleep quality were observed between night shift work schedules (*P*  <  0.05). According to the chi-square test, night shift work schedule was associated with subjective sleep quality, sleep latency, and sleep efficiency when a score of 2 was the cutoff point for each sleep quality component (*P*  <  0.05; Figure 1(b)).

### 3.5. Associations between Quality of Work Life and Sleep Quality

**Figure 2:**
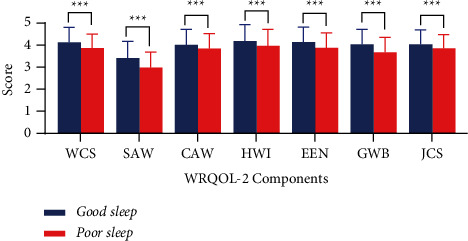
Quality of work life on good and poor sleep groups (*n* = 1426). Error bar: mean ± standard deviations. ^*∗∗∗*^*P*  < 0.001. WSC: working conditions; SAW: stress at work; CAW: control at work; HWI: home-work interface; EEN: employment evaluation of nurse; GWB: general well-being; JCS: job and career satisfaction.

The mean (SD) WRQOL-2 scores for the good and poor sleep groups were 3.98 (SD = 0.56) and 3.70 (SD = 0.55), respectively. In terms of the seven parts of WRQOL-2, the highest scores were for homework interface, while the lowest were for stress at work. The independent samples *t*-test revealed that all dimensions, including working conditions, stress at work, control at work, home-work interface, evaluation at work, general well-being, and job career, were significantly different between the good and poor sleep groups (*P*  <  0.001; Figure 2).

**Figure 3:**
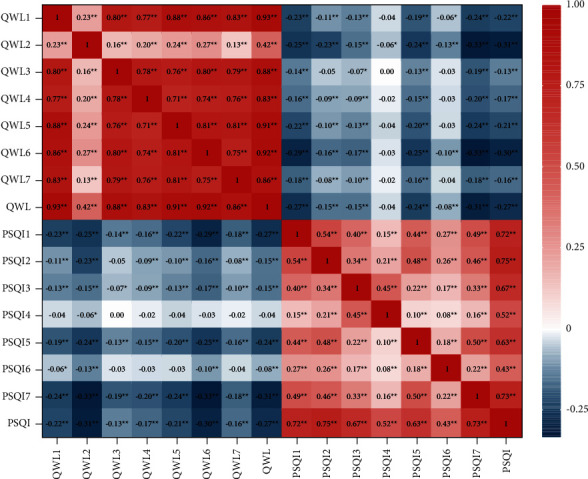
Heat map of Pearson correlation between quality of work life and sleep quality among MCH nurses (*n* = 1426). ^*∗*^*P* < 0.05. ^*∗∗*^*P* < 0.01. PSQI: the Pittsburgh Sleep Quality Index, PSQI1: subjective sleep quality, PSQI2: sleep latency, PSQI3: sleep duration, PSQI4: sleep efficiency, PSQI5: sleep disturbance, PSQI6: sleep medication, and PSQI7: daytime dysfunction; QWL: the Work-Related Quality of Life-2 scale, QWL1: working conditions (WCS), QWL2: stress at work (SAW), QWL3: control at work (CAW), QWL4: home-work interface (HWI), QWL5: employment evaluation of nurse (EEN), QWL6: general well-being (GWB), and QWL7: job and career satisfaction (JCS).

The Pearson correlation test revealed that quality of work life was negatively correlated with sleep quality (*r* = −0.27, *P*  <  0.01). Specifically, the strongest negative association with quality of work life was found for daytime dysfunction (*r* = −0.31, *P*  <  0.01), followed by subjective sleep quality (*r* = −0.27, *P*  <  0.01) and sleep disturbance (*r* = −0.24, *P*  <  0.01). Quality of work life was weakly correlated with sleep latency (*r* = −0.15, *P*  <  0.01), sleep duration (*r* = −0.15, *P*  <  0.01), and sleep medication (*r* = −0.08, *P*  <  0.01). No significant correlation difference was observed between sleep efficiency and quality of work life (*r* = −0.04, *P* = 0.181). The correlation heat map is shown in Figure 3.

### 3.6. Binary Logistic Regression Analysis for Poor Sleep Quality

The binary logistic regression analysis for sleep quality is presented in Table 3 (good sleep = 0; poor sleep = 1). The ORs (95% CI) for the intermediate-morning, intermediate-evening, and evening chronotypes (reference: morning chronotype) were 1.87 (1.20–2.93), 3.61 (2.37–5.50), and 6.70 (4.08–11.01), respectively, in the crude model. In the adjusted model (adjusted for age, educational level, duration of mobile phone use before bedtime, frequency of irregular meals, frequency of caffeine intake, personal monthly income, department, professional rank, and employment type), chronotype remained statistically associated with sleep quality. Regarding night shift work schedules, the ORs (95% CI) for the backward-rotating and 12-hour rotating shifts (reference: day shift) were 1.36 (1.06–1.75) and 1.54 (1.11–2.15), respectively, in the crude model. However, no statistically significant differences were observed based on night shift work schedule for sleep quality in the adjusted model. Significant differences were observed across all dimensions of quality of work life in the crude model; however, the adjusted factors modified the association between quality of work life and sleep quality. The adjusted ORs (95% CI) for stress at work, control at work, and general well-being were 0.53 (0.45–0.63), 2.00 (1.39–2.89), and 0.31 (0.21–0.48), respectively. Working conditions, home-work interface, employment evaluation of nurses, and job and career satisfaction were not significant factors in the adjusted model.

Among the adjusted factors, age, frequency of irregular meals, and frequency of caffeine intake were associated with poor sleep quality. The adjusted ORs (95% CI) for ages 36–45 and ≥46 (reference: age ≤25) were 2.31 (1.23–4.34) and 4.14 (1.56–10.97), respectively. The ORs (95% CI) for irregular meals sometimes, often, and always (reference: never) were 2.73 (1.87–3.98), 4.74 (3.03–7.42), and 7.02 (3.08–16.01), respectively. For caffeine intake frequency, the OR (95% CI) for always (reference: sometimes) was 1.65 (1.12–2.41). For more details, see Supplemental Table S1 (logistic regression analysis for poor sleep quality).

## 4. Discussion

This study revealed a concerningly high rate of poor sleep quality among MCH nurses (57.9%), which was higher than that of nurses in the community (46%) [35], psychiatric hospitals (38.1%) [36], and general hospitals (55%) [16]. This may be due to differences in workplaces and workflows between healthcare organisations, as MCH nurses have to deal with challenging situations such as inappropriate children, overbearing families, noisy workplaces, excessive workloads, a high risk of workplace violence, and emotionally traumatic events [19, 37–39]. Additionally, most of the MCH nurses in this study were female (98.2%). Due to their physiological characteristics, social struggles, and household responsibilities, female nurses experience more psychological distress and sleep problems [40]. These factors may disrupt their circadian rhythms and produce stress that acts on the hypothalamus-pituitary-adrenal axis, increasing the likelihood of sleep problems [41]. In summary, evidence-based strategies for sleep optimisation should be adopted [42].

The current study demonstrated that chronotype may be related to MCH nurses' sleep quality. MCH nurses with evening chronotypes were approximately four times more likely to experience poor sleep than those with morning chronotypes, which is similar to the findings of previous studies [25, 43, 44]. Individuals with evening chronotypes reported chronic disruption of their circadian rhythms and were more susceptible to light-at-night disturbance of melatonin secretion [45]. MCH nurses with evening chronotypes tend to stay up and sleep late and find it difficult to naturally fall asleep early the night before a workday, resulting in sleep deprivation and exhaustion during the workday. Conversely, if they deliberately advance their bedtime to acclimate themselves to their work schedule, it will result in difficult sleep initiation and lower sleep efficiency. Moreover, people with evening chronotypes prefer having snacks at night, which may further delay bedtime and impair subjective sleep quality [46]. Therefore, managers should attempt to create shift schedules in accordance with the natural chronotypes of MCH nurses to increase their sleep quality and quantity.

Univariate analysis revealed an association between night shift work schedule and poor sleep quality. However, this effect was not significant after adjusting for related factors, which may be related to the uneven distribution of various shift types, with nearly half of the participants working backward-rotating night shifts. Nevertheless, shift work is a crucial component of nurses' work lives. Several studies have demonstrated that shift work causes a misalignment of circadian rhythms, and the body's sensitivity to the sleep-wake cycle is diminished, disrupting the rhythm of cortisol and melatonin and leading to sleep problems [47]. Similar to previous studies [48, 49], our study found that participants on a 12-hour rotating night shift showed the highest rates of poor sleep quality, and those on forward-rotating night shifts had better sleep quality than those on either 12-hour rotating or backward-rotating night shifts. As an ergonomic and health-promoting option, a forward-rotating night shift is better adapted to sleep because the human circadian sleep-wake cycle physiology tends to move forward [50, 51]. Consequently, a forward-rotating night shift is recommended to optimise the rotation.

The MCH nurses in this study reported a moderately high level of quality of work life. The stress at work dimension had the lowest score, indicating that MCH nurses typically work under severe stress. Excessive work stress is one contributor to sleep problems, which is consistent with our finding of a high rate of poor sleep. Furthermore, high control at work is a risk factor for poor sleep. Control at work reflects the ability to be competent in nursing and decision-making latitude in the workplace [52]. MCH nurses with higher control at work have difficulty disengaging from the high level of work alertness and repeatedly think about today's work or worry about that of tomorrow even when off duty [53, 54]. This prevents them from fully relaxing and impairs their sleep. In contrast, MCH nurses' general well-being was a protective factor that supported healthy sleep. General well-being is an important component of quality of work life, reflecting satisfaction with overall work life. Good well-being promotes physical and mental health and helps individuals take action to cope with stress and enjoy good sleep [55]. Thus, these findings offer a new perspective on promoting good sleep quality through interventions (e.g., mindfulness and cognitive-behavioural therapy) to improve quality of life at work.

Older age, frequent caffeine consumption, and irregular meals were also associated with poor sleep quality in this study. MCH nurses over 35 years of age were more likely to experience poor sleep quality. With respect to biology, aging causes sleep to become fragmented, and older persons may awaken several times during the night [56]. In terms of work, older nurses with rich work experience take on greater responsibilities, take on heavier nursing workloads, face critical patients and emergencies, and supervise junior nurses, all of which expend more energy and lead to poor sleep. Notably, to induce wakefulness on duty and optimise alertness off duty, MCH nurses as shift worker may become dependent on caffeine because it increases alertness in a dose-dependent manner [57, 58]. Consequently, many of these nurses regularly consume caffeine and experience sleep disturbance. Consistent with previous research [23], irregular meal habits were found to be a risk factor for poor sleep quality. Dietary factors influence or modulate neurotransmitter mechanisms in the brain, which may have downstream effects on sleep [59]. Furthermore, poor diet quality and quantity may affect tryptophan availability and serotonin and melatonin synthesis, leading to poor sleep quality [60]. Thus, these factors are worth considering when in relation to altering sleep quality.

### 4.1. Limitations

The present study has a few limitations. First, the study was conducted among nurses working in four MCH hospitals in one province of China, which may not be representative of all Chinese MCH nurses. Second, owing to the cross-sectional design of this study, the analysis may explain the sleep quality level at a certain point in time. Third, the data were self-reported rather than objectively measured (e.g., actigraphy), which may have introduced subjectivity and bias.

## 5. Conclusions

This study showed that poor sleep quality was common among MCH nurses. The findings of this study also illustrated that sleep quality was associated with older age, frequent caffeine intake, irregular meals, chronotype, and quality of work life. These findings have substantial implications for future studies and policy interventions to enhance MCH nurses' sleep quality and well-being.

## 6. Implications for Nursing Management

This study provides new insights into improving sleep quality to enhance quality of work life and professional satisfaction in the nursing management sector of MCH hospitals. To target current modifiable factors, it is critical to establish a scientific shift work schedule that meets the physical and psychological needs of MCH nurses, ensures adequate rest, and maintains a balance between family and work. In addition, nursing managers can form psychological support teams to provide professional counselling and intervention services for MCH nurses. Accurate sleep information, such as maintaining a balanced diet, avoiding large meals before bedtime, and limiting caffeine to 6 h before sleep, can be conveyed to MCH nurses through brochures or lectures. However, to target nonmodifiable factors, nursing managers should allocate human resources appropriately according to chronotype and age. For example, nurses with morning chronotypes should be assigned to early shifts, whereas those with evening chronotypes should have a delayed start time for their work. As older nurses' energy and fitness levels decline, their night shifts should be reduced and their supervisory and teaching responsibilities should be increased. Night shifts could be added for young nurses to help them develop their clinical skills while increasing their salaries.

## Figures and Tables

**Table 1:** Demographic characteristics of participants (*n* = 1426).

Variables	*N* (%)/*M* *±* SD			*t*/*x*^2^	*p*
	Overall (*n* = 1426)	Good sleep (*n* = 600)	Poor sleep (*n* = 826)		
Age (years)	30.20 ± 7.60	29.66 ± 7.71	30.59 ± 7.50	−2.266	**0.024**
Body mass index (kg/m^2^)	20.95 ± 2.70	21.03 ± 2.75	20.89 ± 2.67	0.941	0.347
Sex				3.354	0.067
Female	1401 (98.2)	585 (97.5)	816 (98.8)		
Male	25 (1.8)	15 (2.5)	10 (1.2)		
Educational level				6.152	**0.046**
Technical secondary school degree	76 (5.3)	37 (6.2)	39 (4.7)		
Junior college degree	754 (52.9)	334 (55.7)	420 (50.8)		
Bachelor degree and above	596 (41.8)	229 (38.2)	367 (44.4)		
Marital status				0.214	0.644
Unmarried	660 (46.3)	282 (47.0)	378 (45.8)		
Married	766 (53.7)	318 (53.0)	448 (54.2)		
Number of children				0.017	0.992
0	725 (50.8)	304 (50.7)	421 (51.0)		
1	387 (27.1)	163 (27.2)	224 (27.1)		
≥2	314 (22.0)	133 (22.2)	181 (21.9)		
Duration of mobile phone use before bedtime				6.936	**0.031**
<1 hour	411 (28.8)	194 (32.3)	217 (26.3)		
1–3 hours	753 (52.8)	307 (51.2)	446 (54.0)		
>3 hours	262 (18.4)	99 (16.5)	163 (19.7)		
Frequency of irregular meals				130.179	<**0.001**
Never	212 (14.9)	152 (25.3)	60 (7.3)		
Sometimes (1-2 times/week)	821 (57.6)	354 (59.0)	467 (56.5)		
Often (3–5 times/week)	339 (23.8)	83 (13.8)	256 (31.0)		
Always (6-7 times/week)	54 (3.8)	11 (1.8)	43 (5.2)		
Frequency of caffeine intake				15.669	<**0.001**
Sometimes (0–2 times/week)	376 (26.4)	182 (30.3)	194 (23.5)		
Often (3–5 times/week)	787 (55.2)	332 (55.3)	455 (55.1)		
Always (6-7 times/week)	263 (18.4)	86 (14.3)	177 (21.4)		
Frequency of sugar intake				3.923	0.141
Sometimes (0–2 times/week)	301 (21.1)	141 (23.5)	160 (19.4)		
Often (3–5 times/week)	972 (68.2)	400 (66.7)	572 (69.2)		
Always (6-7 times/week)	153 (10.7)	59 (9.8)	94 (11.4)		
Physical activity				0.067	0.967
Low	182 (12.8)	75 (12.5)	107 (13.0)		
Moderate	634 (44.5)	268 (44.7)	366 (44.3)		
High	610 (42.8)	257 (42.8)	353 (42.7)		
Personal monthly income (yuan)				18.278	**0.001**
<3000	38 (2.7)	22 (3.7)	16 (1.9)		
3000–5999	386 (27.1)	183 (30.5)	203 (24.6)		
6000–8999	530 (37.2)	216 (36.0)	314 (38.0)		
9000–11999	340 (23.8)	117 (19.5)	223 (27.0)		
≥12000	132 (9.3)	62 (10.3)	70 (8.5)		
Department				15.175	**0.034**
Delivery room	106 (7.4)	38 (6.3)	68 (8.2)		
Obstetrics	239 (16.8)	113 (18.8)	126 (15.3)		
Gynaecology	230 (16.1)	97 (16.2)	133 (16.1)		
Emergency	156 (10.9)	57 (9.5)	99 (12.0)		
Clinic	140 (9.8)	72 (12.0)	68 (8.2)		
Operating room	136 (9.5)	60 (10.0)	76 (9.2)		
Intensive care unit	274 (19.2)	100 (16.7)	174 (21.1)		
Pediatrics	145 (10.2)	63 (10.5)	82 (9.9)		
Professional rank				13.212	**0.004**
Junior nurse	584 (41.0)	273 (45.5)	311 (37.7)		
Senior nurse	604 (42.4)	228 (38.0)	376 (45.5)		
Assistant advanced nurse	204 (14.3)	80 (13.3)	124 (15.0)		
Associate advanced nurse or advanced nurse	34 (2.4)	19 (3.2)	15 (1.8)		
Employment type				3.943	**0.047**
Formal employees	474 (33.2)	182 (30.3)	292 (35.4)		
Contract employees	952 (66.8)	418 (69.7)	534 (64.6)		
PSQI global score	7.90 ± 3.67	4.43 ± 1.87	10.42 ± 2.36	−53.43	<**0.001**
Subjective sleep quality	1.44 ± 0.71	0.96 ± 0.52	1.79 ± 0.63	−27.10	<**0.001**
Sleep latency	1.61 ± 0.96	0.90 ± 0.68	2.12 ± 0.79	−31.40	<**0.001**
Sleep duration	1.28 ± 0.83	0.79 ± 0.65	1.64 ± 0.76	−22.77	<**0.001**
Sleep efficiency	0.76 ± 0.93	0.32 ± 0.57	1.08 ± 1.01	−18.12	<**0.001**
Sleep disturbance	1.18 ± 0.66	0.78 ± 0.53	1.47 ± 0.60	−23.11	<**0.001**
Sleep medication	0.21 ± 0.57	0.01 ± 0.13	0.35 ± 0.71	−13.34	<**0.001**
Daytime dysfunction	1.43 ± 1.01	0.68 ± 0.70	1.98 ± 0.84	−30.75	<**0.001**

Bold values indicate that the *p* value is less than 0.05, indicating statistical significance.

**Table 2:** PSQI and its component score among MCH nurses (*n* = 1426).

Components	Group	Scores	*N* (%)
Subjective sleep quality	Very good	0	91 (6.4)
	Fairly good	1	710 (49.8)
	Fairly bad	2	529 (37.1)
	Very bad	3	96 (6.7)

Sleep latency^†^	0	0	187 (13.1)
	1-2	1	487 (34.2)
	3-4	2	451 (31.6)
	5-6	3	301 (21.1)

Sleep duration	Greater than 7 h	0	256 (18.0)
	6-7 h	1	604 (42.4)
	5-6 h	2	476 (33.4)
	Less than 5 h	3	90 (6.3)

Sleep efficiency	Greater than 85%	0	733 (51.4)
	75%–85%	1	407 (28.5)
	65–74%	2	185 (13.0)
	Less than 65%	3	101 (7.1)

Sleep disturbance^‡^	0	0	175 (12.3)
	1–9	1	860 (60.3)
	10–18	2	356 (25.0)
	19–27	3	35 (2.5)

Sleep medication	Not during the past month	0	1222 (85.7)
	Less than once a week	1	133 (9.3)
	Once or twice a week	2	50 (3.5)
	Three or more times each week	3	21 (1.5)

Daytime dysfunction^§^	0	0	296 (20.8)
	1-2	1	475 (33.3)
	3-4	2	396 (27.8)
	5-6	3	259 (18.2)

PSQI global score	Good sleep	≤7	600 (42.1)
	Poor sleep	>7	826 (57.9)

^†^Sleep latency: scored on two items, including how long it takes to fall asleep each night and how often it is difficult falling asleep within 30 minutes. ^‡^Sleep disturbance: scored on nine items, including waking up in the middle of the night or early morning, getting up to use the bathroom, breathing uncomfortably, coughing or snoring loudly, feeling cold, feeling hot, having bad dreams, having pain, and other reason of troubling sleep. ^§^Daytime dysfunction: scored on two items including the frequency of having trouble staying awake and the frequency of keeping up enough enthusiasm.

**Table 3:** Binary logistic regression analysis for poor sleep based on chronotype, night shift work schedule, and quality of work life.

	Crude ORs (95% CI)	*P*	Adjusted^†^ORs (95% CI)	*P*
*Chronotype*				
Morning (reference)	1		1	
Intermediate-morning	1.87 (1.20–2.93)	**0.006**	1.75 (1.05–2.91)	**0.033**
Intermediate-evening	3.61 (2.37–5.50)	**<0.001**	2.70 (1.66–4.40)	**<0.001**
Evening	6.70 (4.08–11.01)	**<0.001**	3.99 (2.24–7.10)	**<0.001**

*Night shift work schedule*				
Non-night shift (reference)	1		1	
Forward-rotating night shift	1.19 (0.77–1.82)	0.434	0.98 (0.54–1.75)	0.939
Backward-rotating night shift	1.36 (1.06–1.75)	**0.017**	1.08 (0.74–1.58)	0.685
12-hour rotating night shift	1.54 (1.11–2.15)	**0.01**	1.23 (0.74–2.02)	0.426

*Quality of work life*				
Working conditions	0.54 (0.45–0.64)	**<0.001**	1.03 (0.63–1.69)	0.893
Stress at work	0.44 (0.37–0.51)	**<0.001**	0.53 (0.45–0.63)	**<0.001**
Control at work	0.70 (0.60–0.82)	**<0.001**	2.00 (1.39–2.89)	**<0.001**
Home-work interface	0.66 (0.57–0.77)	**<0.001**	1.10 (0.81–1.48)	0.541
Employment evaluation of nurse	0.56 (0.47–0.66)	**<0.001**	1.23 (0.82–1.85)	0.307
General well-being	0.45 (0.38–0.53)	**<0.001**	0.31 (0.21–0.48)	**<0.001**
Job and career satisfaction	0.63 (0.53–0.75)	**<0.001**	0.75 (0.50–1.11)	0.152

^†^Adjusted for age, educational level, duration of mobile phone use before bedtime, frequency of irregular meals, frequency of caffeine intake, personal monthly income, department, professional rank, and employment type. ORs: odds ratios; CI: confidence interval. Bold values indicate that the *p* value is less than 0.05, indicating statistical significance.

## Data Availability

The data used to support the findings of this study are available from the corresponding author upon request.

## Supplementary Materials

Supplementary Materials Supplemental Table S1: logistic regression analysis for poor sleep quality.
