# Supplementary material for: Exploring the Associations between Chronotype, Night Shift Work Schedule, Quality of Work Life, and Sleep Quality among Maternal and Child Health Nurses: A Multicentre Cross-Sectional Study
Source: J Nurs Manag. 2023 Sep 30;2023:1811732. doi: 10.1155/2023/1811732 (PMC11918974; doi:10.1155/2023/1811732)
Supplement: Supplementary Materials — Supplemental Table S1: logistic regression analysis for poor sleep quality. [file 1811732.f1.docx]

| **Supplemental Table S1 Binary logistic regression analysis for poor sleep quality** | | | | |
| --- | --- | --- | --- | --- |
|  | Crude | *P* | Adjusted | *P* |
|  | ORs (95% CI) |  | ORs (95% CI) |  |
| Age (years) |  |  |  |  |
| ≤25 (reference) | 1 |  | 1 |  |
| 26–35 | 1.28(1.01-1.63) | **0.039** | 1.45(0.90-2.33) | 0.124 |
| 36–45 | 1.48(1.09-2.01) | **0.012** | 2.31(1.23-4.34) | **0.009** |
| ≥46 | 1.12(0.64-1.95) | 0.699 | 4.14(1.56-10.97) | **0.004** |
| Educational level |  |  |  |  |
| Technical secondary school degree (reference) | 1 |  | 1 |  |
| Junior college degree | 1.19(0.74-1.91) | 0.464 | 0.93(0.51-1.70) | 0.82 |
| Bachelor degree and above | 1.52(0.94-2.45) | 0.087 | 0.93(0.48-1.78) | 0.817 |
| Duration of mobile phone use before bedtime | |  |  |  |
| ＜1 hour (reference) | 1 |  | 1 |  |
| 1-3 hours | 1.30(1.02-1.65) | **0.034** | 1.20(0.89-1.62) | 0.224 |
| ＞3 hours | 1.47(1.07-2.02) | **0.017** | 1.17(0.78-1.74) | 0.449 |
| Frequency of irregular meals |  |  |  |  |
| Never (reference) | 1 |  | 1 |  |
| Sometimes (1-2 times/week) | 3.34(2.40-4.64) | **＜0.001** | 2.73(1.87-3.98) | **＜0.001** |
| Often (3-5 times/week) | 7.81(5.30-11.52) | **＜0.001** | 4.74(3.03-7.42) | **＜0.001** |
| Always (6-7 times/week) | 9.90(4.79-20.48) | **＜0.001** | 7.02(3.08-16.01) | **＜0.001** |
| Frequency of caffeine intake |  |  |  |  |
| Sometimes (0-2 times/week) | 1 |  | 1 |  |
| Often (3-5 times/week) | 1.29(1.00-1.65) | **0.046** | 1.30(0.97-1.75) | 0.079 |
| Always (6-7 times/week) | 1.93(1.39-2.68) | **＜0.001** | 1.65(1.12-2.41) | **0.011** |
| Personal monthly income (yuan) |  |  |  |  |
| <3000 (reference) | 1 |  | 1 |  |
| 3000 - 5999 | 1.53(0.78-2.99) | 0.22 | 1.44(0.64-3.27) | 0.381 |
| 6000 - 8999 | 2.00(1.03-3.89) | **0.042** | 1.86(0.81-4.30) | 0.145 |
| 9000 - 11999 | 2.62(1.33-5.18) | **0.006** | 1.80(0.75-4.32) | 0.188 |
| ≥12000 | 1.55(0.75-3.22) | 0.237 | 1.04(0.40-2.68) | 0.939 |
| Department |  |  |  |  |
| Delivery room (reference) | 1 |  | 1 |  |
| Obstetrics | 0.62(0.39-1.00) | **0.049** | 0.79(0.46-1.36) | 0.395 |
| Gynecology | 0.77(0.48-1.23) | 0.272 | 0.96(0.55-1.68) | 0.886 |
| Emergency | 0.97(0.58-1.62) | 0.909 | 1.31(0.71-2.41) | 0.393 |
| Clinic | 0.53(0.31-0.89) | **0.015** | 0.90(0.46-1.78) | 0.771 |
| Operating room | 0.71(0.42-1.19) | 0.194 | 0.67(0.34-1.33) | 0.254 |
| Intensive care unit | 0.97(0.61-1.55) | 0.906 | 0.86(0.50-1.47) | 0.576 |
| Pediatrics | 0.73(0.43-1.22) | 0.226 | 0.79(0.42-1.50) | 0.478 |
| Professional rank |  |  |  |  |
| Junior nurse (reference) | 1 |  | 1 |  |
| Senior nurse | 1.45(1.15-1.82) | **0.002** | 1.16(0.72-1.87) | 0.534 |
| Assistant advanced nurse | 1.36(0.98-1.88) | 0.063 | 1.10(0.58-2.10) | 0.765 |
| Associate advanced nurse or advanced nurse | 0.69(0.35-1.39) | 0.302 | 0.34(0.12-1.01) | 0.052 |
| Employment type |  |  |  |  |
| Formal employees (reference) |  |  |  |  |
| Contract employees | 0.80(0.64-1.00) | **0.047** | 0.92(0.61-1.38) | 0.67 |
| Chronotype |  |  |  |  |
| Morning (reference) | 1 |  | 1 |  |
| Intermediate - morning | 1.87(1.20-2.93) | **0.006** | 1.75(1.05-2.91) | **0.033** |
| Intermediate - evening | 3.61(2.37-5.50) | **＜0.001** | 2.70(1.66-4.40) | **＜0.001** |
| Evening | 6.70(4.08-11.01) | **＜0.001** | 3.99(2.24-7.10) | **＜0.001** |
| Night shift work schedule |  |  |  |  |
| Non-night shift (reference) | 1 |  | 1 |  |
| Forward-rotating night shift | 1.19(0.77-1.82) | 0.434 | 0.98(0.54-1.75) | 0.939 |
| Backward-rotating night shift | 1.36(1.06-1.75) | **0.017** | 1.08(0.74-1.58) | 0.685 |
| 12-hour rotating night shift | 1.54(1.11-2.15) | **0.01** | 1.23(0.74-2.02) | 0.426 |
| Quality of work life |  |  |  |  |
| Working conditions | 0.54(0.45-0.64) | **＜0.001** | 1.03(0.63-1.69) | 0.893 |
| Stress at work | 0.44(0.37-0.51) | **＜0.001** | 0.53(0.45-0.63) | **＜0.001** |
| Control at work | 0.70(0.60-0.82) | **＜0.001** | 2.00(1.39-2.89) | **＜0.001** |
| Homework interface | 0.66(0.57-0.77) | **＜0.001** | 1.10(0.81-1.48) | 0.541 |
| Employment evaluation of nurse | 0.56(0.47-0.66) | **＜0.001** | 1.23(0.82-1.85) | 0.307 |
| General well-being | 0.45(0.38-0.53) | **＜0.001** | 0.31(0.21-0.48) | **＜0.001** |
| Job and career satisfaction | 0.63(0.53-0.75) | **＜0.001** | 0.75(0.50-1.11) | 0.152 |

ORs: Odds ratios; CI: confidence interval.
